# BAD NEWS FOR HYPOCHONDRIACS: Covid-19 Associated Aggravation of Somatic Symptom Disorder with Psychotic Depression

**DOI:** 10.1192/j.eurpsy.2022.1005

**Published:** 2022-09-01

**Authors:** S. Jesus, A. Costa, G. Simões, P. Garrido

**Affiliations:** Baixo Vouga Hospital Centre - EPE, Psychiatry And Mental Health Department, Aveiro, Portugal

**Keywords:** Psychotic depression, covid 19, somatoform

## Abstract

**Introduction:**

The Covid-19 pandemic has brought with it far-reaching consequences that affect the mental health of a significant population. Those suffering from somatic symptom disorder (SSD) present a significant focus on physical symptoms, with excessive thoughts and behaviours, to an extent that results in major distress and dysfunction. Aggravation of SSD could be associated with various stressors, including the current pandemic, and culminate in an increased severity of the base presentation.

**Objectives:**

The authors present the case of an elderly man with previous diagnosis of SSD which began to aggravate and evolve into a depressive psychotic state, precipitated by the beginning of the Covid-19 pandemic.

**Methods:**

The authors conducted a non-systematized literature review with focus on those articles most pertinent to the topic in question as well as presenting a clinical case as compliment.

**Results:**

With the pandemic overwhelming the globe, the literature has demonstrated a significant correlation with aggravation of mental health and psychiatric cases. The patient in question was previously followed in consultation for SSD. With the pandemic acting as precipitating stressor, the patient demonstrated a significant aggravation in his base presentation with the development of psychotic depression. He was subsequently hospitalized with implementation of psychotherapeutic and psychopharmacological methods, with remission of the psychotic state, with poor response of the SSD.

**Conclusions:**

External stressors are an important influence on psychiatric disorders. Whenever potential life stressors, especially those that exert influence on a global scale, the psychiatrist should be attentive to the possibility of significant aggravation of a stabilized clinical picture and offer support.

**Disclosure:**

No significant relationships.

